# Age cohorts stratified according to age-distributions of COVID-19 morbidity statistics identify uniquely age-dependent CD3^+^CD8^+^ T-cell lymphocytopenia in COVID-19 patients without comorbidities on admission

**DOI:** 10.18632/aging.202691

**Published:** 2021-03-10

**Authors:** Shengwei Jin, Hui An, Tong Zhou, Ting Li, Chengshui Chen, Binyu Ying, Zhangye Xu, Xiaokun Li, Ming Li

**Affiliations:** 1 School of Basic Medical Science, Wenzhou Medical University, Wenzhou, Zhejiang, China; 2 Department of Anesthesia and Critical Care, The Second Affiliated Hospital and Yuying Children’s Hospital of Wenzhou Medical University, Wenzhou, Zhejiang, China; 3 Department of Gynecology and Obstetrics, The Second Affiliated Hospital of Wenzhou Medical University, Wenzhou, Zhejiang, China; 4 Department of Pulmonary and Critical Care Medicine, The First Affiliated Hospital of Wenzhou Medical University, Wenzhou, Zhejiang, China; 5 Department of Critical Care Medicine, The Second Affiliated Hospital and Yuying Children's Hospital of Wenzhou Medical University, Wenzhou, Zhejiang, China; 6 School of Pharmacy, Wenzhou Medical University, Wenzhou, Zhejiang, China

**Keywords:** coronavirus disease 2019, COVID-19, age-dependent immune features, CD8+ T cells, C-reactive protein

## Abstract

If age boundaries are arbitrarily or roughly defined, age-related analyses can result in questionable findings. Here, we aimed to delineate the uniquely age-dependent immune features of coronavirus disease 2019 (COVID-19) in a retrospective study of 447 patients, stratified according to age distributions of COVID-19 morbidity statistics into well-defined age-cohorts (2–25y, 26–38y, 39–57y, 58–68y, and 69–79y). Age-dependent susceptibilities and severities of the disease were observed in COVID-19 patients. A comparison of the lymphocyte counts among the five age-groups indicated that severe acute respiratory syndrome coronavirus 2 (SARS-CoV-2) infection led to age-dependent lymphopenia. Among the lymphocyte subsets, the CD8^+^ T cell count alone was significantly and age-dependently decreased (520, 385, 320, 172, and 139 n/μl in the five age-groups, respectively). In contrast, the CD4^+^ T cell, B cell, and natural killer cell counts did not differ among age-cohorts. Age and CD8^+^ T cell counts (r=‒0.435, p<0.0001) were negatively correlated in COVID-19 patients. Moreover, SARS-CoV-2 infection age-dependently increased the plasma C-reactive protein concentrations (2.0, 5.0, 9.0, 11.6, and 36.1 mg/L in the five age-groups, respectively). These findings can be used to elucidate the role of CD8^+^ T cells in age-related pathogenesis and to help develop therapeutic strategies for COVID-19.

## INTRODUCTION

The ongoing coronavirus disease 2019 (COVID-19) pandemic has caused a disproportionate mortality in the older population. Differences in its severity among children, adults, and elders have been observed during the early stages of the pandemic [1–3]. While severe acute respiratory syndrome coronavirus 2 (SARS-CoV-2) infection did not alter blood lymphocyte (including T cells and B cells) levels in both mild and moderate COVID-19 cases in children [4], lymphocytes, CD4^+^ T cells, CD8^+^ T cells, and natural killer (NK) cells were found to be significantly decreased in adults with moderate and severe COVID-19 [3, 5, 6]. Previous age-subgroup analyses of the immune features of COVID-19 focused primarily on roughly defined age boundaries (children, adults, and elders) in a small number of cases. If age boundaries are arbitrarily or roughly defined, age-related analyses can result in inconsistent research findings. In addition, the comorbidities of COVID-19 patients or medical interventions implemented can severely influence its clinical and immunological manifestations. Understanding the underlying mechanisms of less severe COVID-19 in children and young people can help in the development of therapeutic targets for high-risk and older adults; thus, further research on the immune-pathogenesis of COVID-19 in well-defined age-groups is required.

The demographic distribution of COVID-19 incidence is age-dependent. In February 2019, the Chinese Center for Disease Control and Prevention reported a summary of the characteristics of 44,672 confirmed COVID-19 patients from December 2019 to February 2020 of the pandemic. An age-specific bell-shaped incident curve of COVID-19 cases (≥0 ‒ ≤20 years: 0.9 ‒ 8.1%, ≥30 ‒ ≤60 years: 17.0 – 19.2%, ≥80 years: 3.2 %) and an age-dependent linear increase of crude death rate (CDR) were observed [7]. Since COVID-19 detection assays were not performed in patients with no or mild symptoms in the very early stages of the outbreak, this demographic distribution of confirmed COVID-19 cases and CDRs from the earliest stages of the pandemic could have been the result from both age-varying susceptibility and age-varying clinical symptoms. Recently, Davies et al. [8] established an age-stratified transmission model with heterogeneous contact rates between age groups and found that both age-varying susceptibility and age-varying clinical manifestations could have contributed in part to the observed age patterns. This was consistent with the reports that both age-varying susceptibility to SARS-CoV-2 infection [9] and age-varying severity [1] in COVID-19 cases were observed in China. Further studies have also pointed it out that the lower COVID-19 incident rate in older patients was as a result of the lower composition of older age in the general public of China. The COVID-19 incidence risk is low in children but increases in older groups of the general public [10]. Thus, we could speculate that well-defined age boundaries of COVID-19 morbidity may reflect uniquely age-dependent immune features among different age populations. In this study, we investigated the age-dependent immune features of well-defined age-cohorts stratified according to the rule of grouping neighboring ages with similar COVID-19 incident rates.

## RESULTS

### Patient demographics and age- and COVID-19 incidence-specific subgroup characteristics of COVID-19

As of March 20, 2020, a total of 693 (323 women and 370 men) confirmed COVID-19 patients admitted to 10 hospitals in Wenzhou City, Zhejiang Province were enrolled in this study. Of 693 patients, 246 (35.5%) patients had previously been diagnosed with comorbidities, including cardiovascular and cerebrovascular diseases, respiratory system diseases, malignant tumor, chronic liver and kidney diseases, hypercholesterolemia, diabetes mellitus, and others. Out of 693 cases, approximately six percent (5.6%) of cases presented with a mild disease, 78.9% a moderate one, 12.3% a severe one, and 3.2% a critical ill one. By the data collection deadline, all patients have been discharged. The mean age at COVID-19 onset was 47 years (interquartile range [IQR] 37–56). Almost 53% (53.4%) of the cases were men. After the diagnosis of COVID-19 was confirmed, all patients received antiviral, herbal and supportive therapies.

**Figure 1:**
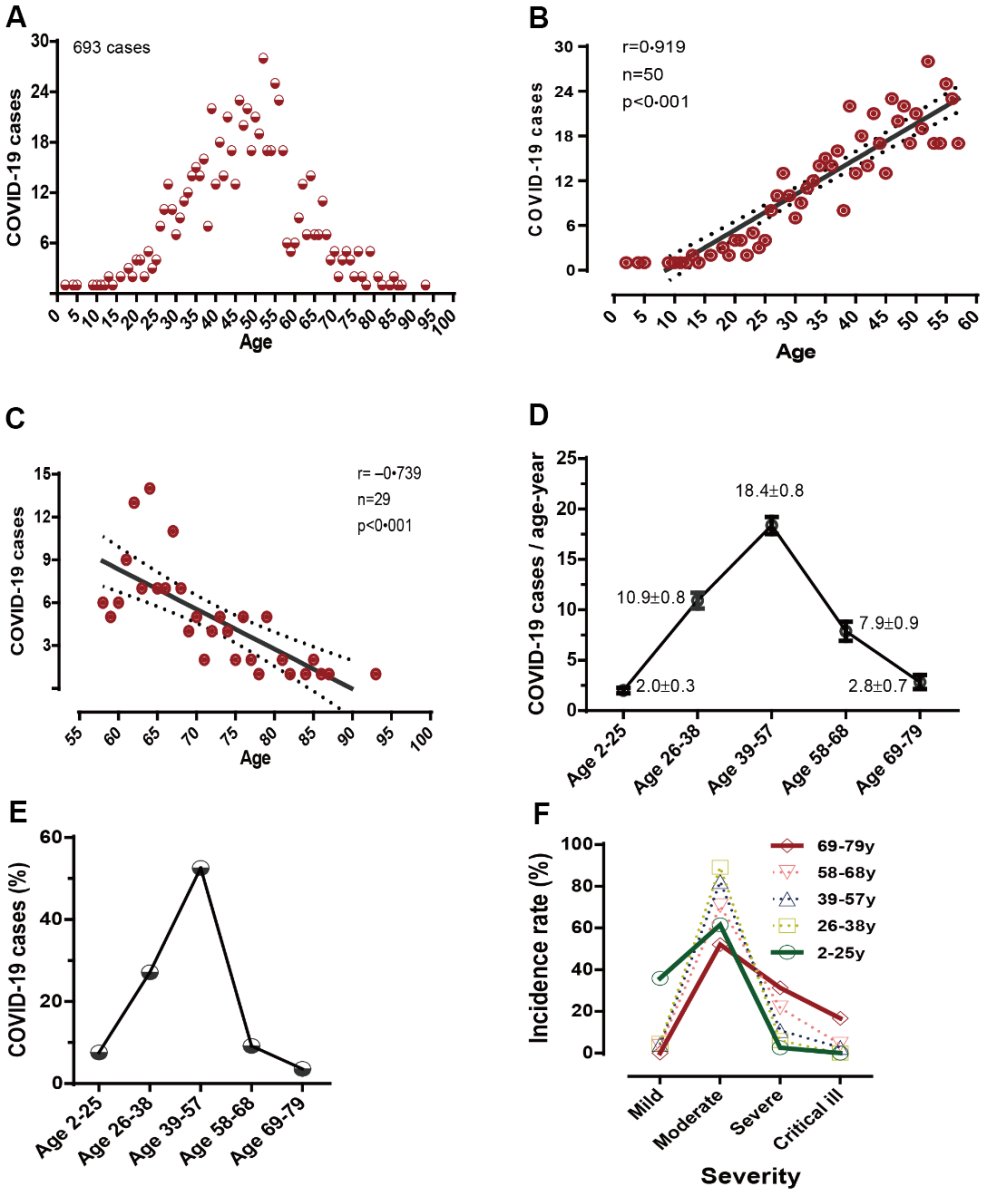
**The distribution of COVID-19 morbidities and disease severities in different age categories.** (**A**) Detailed distributions of COVID-19 morbidities/age-year, (**B**, **C**) correlations between different age and COVID-19 cases/age-year, (**D**) mean values of COVID-19 cases/age-cohort, (**E**) COVID-19 incidence rate/age-cohort, and (**F**) disease severities in different age categories.

To optimally understand how the clinical and immunological characteristics evolved in COVID-19 according to an age-dependence, the appropriate categorization of chronological ages was considered. In this study, we used the time interval of 1-year as the age interval to count the confirmed COVID-19 cases. A graphic plot of the number of COVID-19 cases per year vs age produced a bell-shaped curve (Figure 1A). There was a strong positive correlation between the number of confirmed COVID-19 cases and different year-cohorts in the age range of 2 to 57 years (r=0.919, p<0.001; Figure 1B). In contrast, a negative correlation between the number of confirmed COVID-19 cases and different year-cohorts in the age range of 58 to 93 years was observed (r= ‒0.739, p<0.002; Figure 1C).

To differentiate COVID-19 patients who were previously healthy from those with comorbidities, which showed strong interference in COVID-19 manifestations, data from 447 patients without comorbidities on admission were included in the analysis. Different age-cohorts were combined with their neighboring age cohorts with similar incidence counts/year together. Accordingly, five age-groups were formed as follows: 2‒25y (2.0±0.3 cases/year-cohort, n=34), 26‒38y (10.9±0.8 cases/year-cohort, n=121), 39‒57y (18.4±0.8 cases/year-cohort, n=235), 58‒68y (7.9±0.9 cases/year-cohort, n=41), and 69‒79y (2.8±0.7 cases/year-cohort, n=16) groups (Figure 1D). The COVID-19 incidences in the five age-groups indicated a triangle-shape trend as follows: 0‒25y (7.6%) <26‒38y (27.1%) <39‒57y (52.6%) >58‒68y (9.2%) >69‒79y (3.6%) (Figure 1E and Table 1, all p < 0.001 “<” or “>” p<0.001 between groups).

**Table 1:** Clinical and laboratory profiles of 447 COVID-19 patients with different age cohorts.

		**All patients**	**2-25y**	**26-38y**	**p values**	**39-57y**	**p values**	**58-68y**	**p values**	**69-79y**	**p values**	**p values**
	**Reference ranges**	**(n=447)**	**(n=34)**	**(n=121)**		**(n=235)**		**(n=41)**		**(n=16)**		
**Demographics**												
Age, years		44 (34, 53)	20 (13, 23)	33 (29, 36)	^a^	48 (43, 53)	^aaaa/bbbb^	62 (61, 65)	^aaaa/bbbb/cccc^	73 (69, 76)	^aaaa/bbbb/cccc/dddd^	<0.0001
Incidence, %			34/447 (7.6)	121/447 (27.1)	^a^	235/447 (52.6)	^aaaa/bbbb^	41/447 (9.2)	^bbbb/cccc^	16/447 (3.6)	^aa/bbbb/cccc/ddd^	
Gender, Male %		230/447 (51.5)	23/34 (67.6)	55/121 (45.5)	^a^	127/235 (54.0)		16/41 (39.0)	^a^	9/16 (56.3)		
**Severity**												
Mild		26/447 (5.8)	33/34 (97.1)	7/121 (5.8)	^a^	8/235 (3.4)	^a^	2/41 (4.9)	^a^	0/16 (0)	^a^	
Moderate		377/447 (84.3)	0/34 (0)	109/121 (90.1)	^a^	204/235 (86.8)	^a^	25/41 (61.0)	^aaaa/bbbb/cccc^	10/16 (62.5)	^aaaa/bb/cc^	
Severe		35/447 (7.8)	1/34 (2.9)	5/121 (4.1)		15/235 (6.4)		13/41 (31.7)	^aa/bbbb/cccc^	4/16 (25.0)	^a/bb/cc^	
Critical ill		9/447 (2.0)	0/34 (0)	0/121 (0)		6/235 (2.6)		1/41 (2.4)		2/16 (12.5)	^c^	
Oxygen index, mmHg	>300 mmHg	424 (355, 486)	462 (403, 486)	461 (396, 500)		416 (352, 463)	^b^	386 (271, 469)	^b^	379 (282, 486)		
<300 mmHg		39/354 (11.0)	1/19 (5.3)	5/93 (5.4)		17/190 (9.0)		12/37 (32.4)	^a/bbbb/cccc^	4/15 (26.7)	^bb/c^	
**Laboratory parameters**												
Leukocyte count, ×10^9^ /L	3.5-9.5 ×10^9^ /L	4.8 (3.8, 6.2)	5.3 (4.3, 6.5)	4.7 (3.5, 6.0)		4.7 (3.8, 6.0)		5.6 (4.4, 7.5)		5.4 (4.3, 8.0)		0.0077
>10 ×10^9^ /L		189/438 (43.2)	7/33 (21.2)	35/116 (30.2)		111/232 (47.8)	^aa/bb^	22/41 (53.7)	^aa/bb^	14/16 (87.5)	^aaaa/bbbb/cc/d^	
Neutrophil count, ×10^9^ /L	1.8-6.3 ×10^9^ /L	2.9 (2.1, 4.1)	2.8 (2.3, 4.0)	2.8 (1.8, 3.9)		2.9 (2.1, 3.9)		3.9 (2.6, 5.3)	^bb/cc^	3.5 (2.5, 6.0)		0.0014
>6.3 ×10^9^ /L		30/442 (6.8)	1/33 (3.0)	4/119 (3.4)		15/233 (6.4)		7/41 (17.1)	^bb/c^	3/16 (18.8)	^b^	
Lymphocyte count, ×10^9^ /L	1.1–3.2 ×10^9^ /L	1.3 (1.0, 1.6)	1.6 (1.4, 2.3)	1.3 (1.1, 1.7)		1.3 (1.0, 1.6)		1.0 (0.8, 1.4)		0.9 (0.5, 1.4)		<0.0001
<1.1 ×10^9^ /L		140/443 (31.6)	3/33 (9.1)	30/120 (25.0)	^a^	75/233 (32.2)	^a^	22/41 (53.7)	^aaa/bbb^	10/16 (62.5)	^aaa/bb/c^	
NLR		2.3 (1.5, 3.3)	1.7 (1.0, 2.7)	1.9 (1.4, 2.7)		2.3 (1.5, 3.3)		3.3 (2.6, 5.3)	^aaaa/bbbb/ccc^	3.6 (1.9, 8.1)	^aa/bb^	<0.0001
C-reactive protein, mg/L	<10 mg/L	7.4 (3.5, 21.1)	2.0 (0.8, 9.8)	5.0 (2.2, 12.5)		9.0 (3.9, 24.2)	^aa/bb^	11.6 (5.6, 31.0)	^aa/bb^	36.1 (13.9, 57.2)	^aaaa/bbbb/c^	<0.0001
>10 mg/L		189/438 (43.2)	7/33 (21.2)	35/116 (30.2)		111/232 (47.8)	^aa/bb^	22/41 (53.7)	^aa/bb^	14/16 (87.5)	^aaaa/bbbb/cc/d^	
IL-6, pg/ml	<7 pg/ml	4.0 (2.5, 12.0)	3.8 (3.0, 5.5)	3.5 (2.1, 5.6)		3.8 (2.5, 12.9)		10.3 (4.7, 36.9)	^b^	12.4 (5.4, 50.4)	^b^	0.0028
>7 pg/ml		53/168 (31.6)	2/13 (15.4)	7/41 (17.1)		28/87 (32.2)		10/18 (55.6)	^a/bb^	6/9 (66.7)	^a/bb/c^	
**Lymphocyte subsets**												
Lymphocytes /μl	1100-3200 /μl	1300 (1000, 1600)	1600 (1400, 2300)	1300 (1100, 1700)		1300 (1000, 1600)		1000 (800, 1400)		900 (500, 1400)		<0.0001
<1000 cells /μl		103/443 (23.3)	2/33 (6.1)	17/120 (14.2)		54/233 (23.2)	^a/b^	20/41 (48.8)	^aaaa/bbbb/ccc^	10/16 (62.5)	^aaaa/bbbb/ccc^	
CD4^+ cells^, /μl	550-1440 /μl	518 (356, 693)	601 (414, 781)	523 (405, 588)		534 (363, 755)		398 (247, 533)		378 (209, 690)		0.0585
<300 cells /μl		32/193 (16.6)	0/10 (0)	6/43 (14.0)		16/107 (15.0)		6/20 (30.0)		4/13 (30.8)		
CD8^+^cells /μl	320-1250 /μl	319 (202, 459)	520 (372, 663)	385 (283, 497)	^a^	320 (208, 429)	^a^	172 (94, 313)	^aaa/bbb^	139 (112, 229)	^aaaa/bbb/c^	<0.0001
<200 cells/μl		47/193 (24.4)	0/10 (0)	3/43 (7.0)		24/107 (22.4)	^b^	11/20 (55.0)	^bbbb/cc^	9/13 (69.2)	^bbbb/ccc^	
CD4^+^T /CD8^+^T cell ratio	1.5-2.0	1.6 (1.2, 2.3)	1.1 (0.9, 1.5)	1.3 (1.0, 1.6)		1.8 (1.3, 2.4)	^a/bb^	2.5 (1.5, 3.1)	^aa/bbb^	2.3 (1.9, 3.1)	^aa/bb^	<0.0001
<1.5 ratio		81/193 (42.0)	8/10 (80.0)	30/43 (69.8)		36/107 (33.6)	^aa/bbbb^	5/20 (25.0)	^aa/bbb^	2/13 (15.4)	^aa/bb^	
T cells /μl	955-2860 /μl	859 (570, 1143)	1217 (765, 1410)	917 (682, 1134)		888 (578, 1183)		565 (369, 870)	^a^	497 (343, 919)	^a^	0.001
<500 cells /μl		36/193 (18.7)	0/10 (0)	4/43 (9.3)		16/107 (15.0)		9/20 (45.0)	^a/bb/cc^	7/13 (53.9)	^aa/bbb/ccc^	
B cells /μl	90-560 /μl	165 (123, 237)	246 (157, 266)	138 (123, 209)		168 (124, 235)		164 (101, 265)		169 (150, 218)		0.4295
<90 cells/		10/120 (8.3)	0/8 (0)	1/19 (5.3)		8/67 (11.9)		1/16 (6.3)		0/10 (0)		
NK cells /μl	150-1100 /μl	222 (151, 331)	257 (172, 378)	205 (139, 331)		222 (155, 352)		226 (181, 284)		181 (40, 250)		0.349
<150 cells /μl		28/119 (23.5)	1/8 (12.5)	5/19 (26.3)		15/67 (22.4)		3/15 (20.0)		4/10 (40.0)		

Data are medians (IQRs), n (%), or n/N (%). p values were calculated by Mann-Whitney U test, χ^2^ test, or Fisher’s exact test, as appropriate. NLR=neutrophil to lymphocyte Ratio. IL=interleukin. ^a,b,c,d^ χ^2^ test, Mann-Whitney test, or Fisher’s exact test comparing with 2–25y group, 26–38y group, 39–57y group, or 58–68y group respectively. ^a^p<0.05, ^aa^p<0.01, ^aaa^p<0.001, and ^aaaa^p<0.0001 *vs.* 2–25y group. ^b^p<0.05, ^bb^p<0.01, ^bbb^p<0.001, and ^bbbb^p<0.0001 *vs.* 26–38y group. ^c^p<0.05, ^cc^p<0.01, ^ccc^p<0.001, and ^cccc^p<0.0001 *vs.* 39–57y group. ^d^p<0.05, ^dd^p<0.01, ^ddd^p<0.001, and ^dddd^p<0.0001 *vs.* 58–68y group.

The age differences of the severity between children and older adults had been noticed at the early stages of the COVID-19 pandemic [1]. In the present study, Figure 1F shows an age dependence in severity and critical illness among the five age-groups of COVID-19 patients. Patients in the young age-group 2–25y experienced a mild infection more frequently (97.1%) than those in the older age-groups. The moderate form was found to be significantly associated with the middle age-groups 26–38y (90.1% cases) and 39–57y (86.8% cases). In contrast, more cases with a severe form were associated with the older age-groups 58–68y (31.7%) and 69–79y (25.0%). The critically ill cases in the age-groups 39–57y, 58–68y, and 69–79y accounted for 2.6%, 2.4% and 12.5% respectively.

### Laboratory findings of age- and COVID-19 incidence-specific cohorts on admission

**Figure 2:**
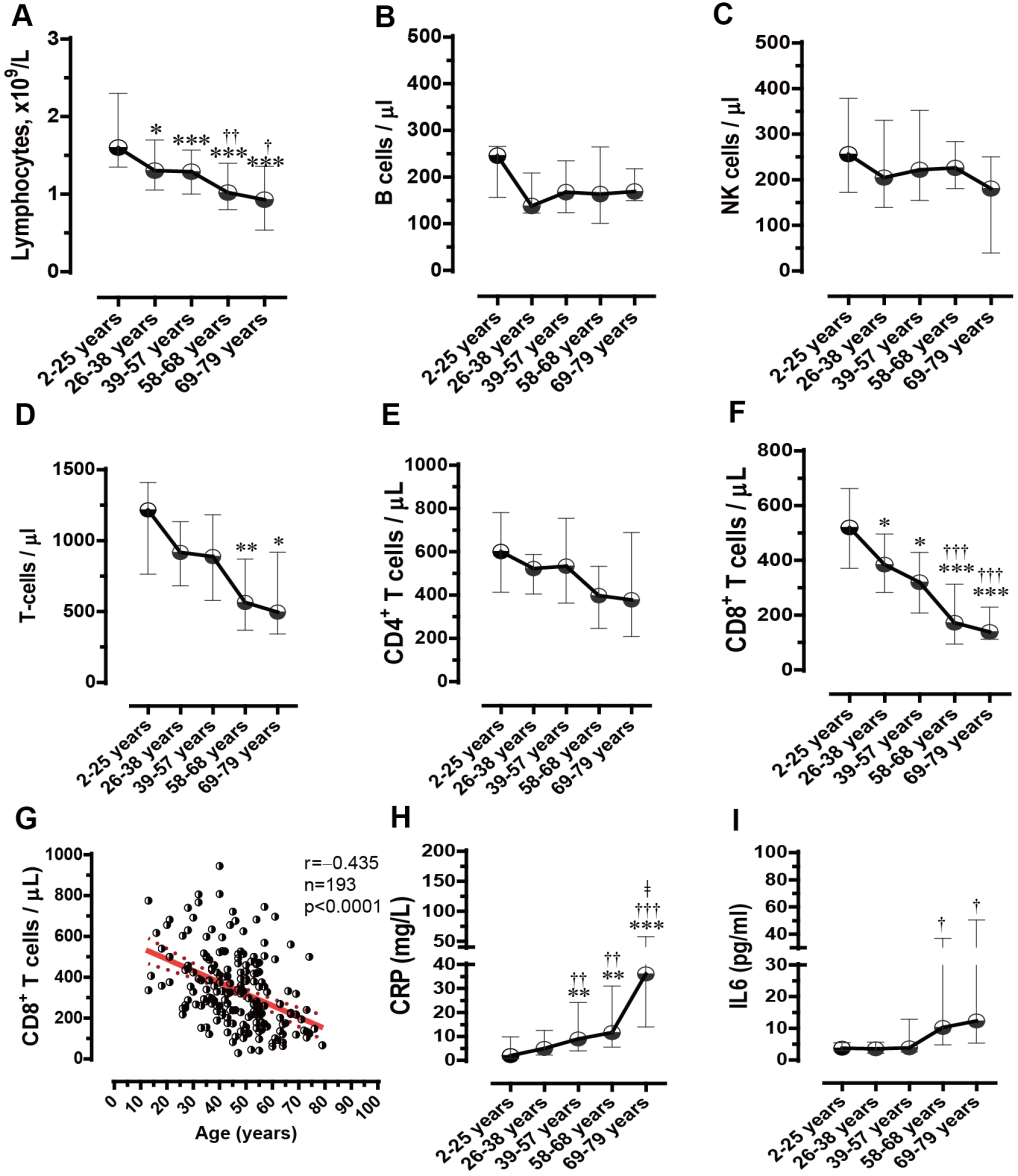
**Blood lymphocyte and subset count, plasma C-reactive protein (CRP) and interleukin 6 (IL-6) levels in COVID-19 patients with different age categories.** (**A**) Lymphocyte counts linearly decreased in the five age groups. (**B**) B cells did not significantly differ among the five age groups. (**C**) Natural killer (NK) cells did not differ among the five age groups. (**D**) Changes in T cell counts in the five age groups. (**E**) CD4^+^ T cells did not significantly differ among the five age groups. (**F**) CD8^+^ T cell counts linearly decreased in the five age groups. (**G**) Correlation between age and CD8^+^ T cell counts. (**H**) CRP levels linearly increased in the five age groups. (**I**) Changes in IL-6 levels in the five age groups. *p<0.05, **p<0.01, and ***p<0.001 *vs.* age group 2–25y; †p<0.05, ††p<0.01, and †††p<0.001 *vs*. age group 26–38y; and ǂ p<0.05 *vs*. age group 39–57y.

From our retrospective analysis of 447 patients on admission, 443 patients had their lymphocyte counts recorded. Laboratory parameters, including leukocyte and neutrophil counts, and plasma C-reactive protein (CRP) and interleukin-6 (IL-6) levels were above the normal reference ranges, in contrast to lymphocyte counts as well as lymphocyte subset counts, which were within the lower part of their ranges (Table 1). Compared with the normal reference ranges for the total lymphocyte count (1.1– 3.2 ×10^9^/L), B cell count (90–560 n/μl), NK cell count (150–1100 n/μl), T cell count (955–2860 n/μl), CD4^+^ T cell count (550–1440 n/μl), CD8^+^ T cell count (320–1250 n/μl), and CD4^+^/CD8^+^ T cell ratio (1.5–2.0), there were 9.1% cases with low lymphocyte counts (<1.1×10^9^/L) and 80% cases with low CD4^+^/CD8^+^ T cell ratio (<1.5) with no significant changes in other cell counts of lymphocyte subsets, in the SARS-CoV-2 infected age-group 2–25y. In the age-group 26–38y, SARS-CoV-2 infection further increased cases with a low lymphocyte count (25.0%, p<0.05) and decreased the CD8^+^ T lymphocyte count (385 cells/μl) when compared with the age-group 2–25y (520 cells/μl, p<0.05). From the age-group 39–57y onwards, the number of cases with lymphopenia (<1.1×10^9^/L) and lower CD8^+^ T cell counts (<200 cells/μl) further linearly increased (Figure 2A) (refer to Table 1 for p values). The decrease in the lymphocytes was not due to B cell and NK cell counts, but due to the T cell counts, as the B cell and NK cell counts did not differ among the age-groups (Figure 2B, 2C); however, T cell counts reduced linearly (Figure 2D). In T cell subpopulations, the CD4^+^ T cell counts did not differ among age-groups (Figure 2E). In contrast, the CD8^+^ T cell counts (n/μl) dropped linearly and sharply from the age-group 26–38y onwards (age-group 2–25y [520 (IQR: 372–663)], 26–38y [385 (IQR:283–497)], 39–57y [320 (IQR: 208–429)], 58–68y [172 (IQR: 94–313)] and 69–79y [139 (IQR: 112–229)], p<0.05, p<0.05, p<0.001 and p<0.001 respectively, Figure 2F). A negative correlation between age and CD8^+^ T cell counts was observed in COVID-19 patients (r=‒0.435, n=193, p<0.0001, Figure 2G), indicating that SARS-CoV-2 infection age-dependently reduces the number of CD8^+^ T cells.

Virus infection is usually associated with systemic inflammatory responses [11]. SARS-CoV-2 infection increased cases of hyper leukemia (>10×10^9^) or high plasma CRP levels (>10 mg/L) both from the age-group 39–57y onwards (Table 1 and Figure 2H). Compared with the age-group 26–38y, plasma IL-6 levels were significantly higher in both the 56–68y and 69–79y groups (Figure 2I, both p<0.05). These results suggest that systemic inflammatory response to SARS-CoV-2 infection are also age-dependent.

## DISCUSSION

As in previous studies, the age differences in the severity between children and older adults have been noticed in the early stages of the COVID-19 pandemic [1]. In adults with COVID-19, total lymphocytes, CD4^+^ T cells, CD8^+^ T cells, and NK cells decreased significantly [3, 5, 6]. In contrast, in children with COVID-19, no statistically significant differences in the absolute number of lymphocyte and its subsets (including T cells and B cells) were observed [4]. However, subgroup analyses with rough age-defined boundaries (children, adults, and older people), theoretically, resulted in conflicting and questionable findings. Since it is difficult to divide chronological ages naturally into immune- and age-specific groups, the true features of the age-dependent immune cell changes of COVID-19 observed thus far, remains to be further clarified.

Here, we delineated the uniquely age-dependent immune features of COVID-19 based on a retrospective study of well-defined age-cohorts stratified according to the age distributions of COVID-19 morbidity statistics. To differentiate COVID-19 patients who were previously healthy from those with comorbidities or medical interventions, which showed strong interferences in COVID-19 manifestations, data from 447 patients without comorbidities on admission were included in the analysis. In this study, we found that SARS-CoV-2 infection could cause a distinctively age-dependent linear reduction in the circulating lymphocytes and T cells, indicating that the age-incidence-dependent division of age groups is tightly associated with the age-dependent immunological response in COVID-19 patients. We noted that both B cells and NK cells were not significantly altered among the five age-groups with COVID-19. This could have been partly due to the direct attack of the T cell [12] by the SARS-CoV-2 virus, thereby producing a more significant decrease in T lymphocytes in COVID-19 patients. The clinical findings that the reduction of T cell counts is strongly associated with the severity of COVID-19 [13] supports this notion. Thus, lymphopenia in COVID-19 attributes mainly to T cells. In the present study, CD8^+^ T cell counts alone decreased in an age-dependent manner, while CD8^+^ T cell showed a tighter negative correlation (r=‒0.435, p<0.001) with age, suggesting that the CD8^+^ T cell subset is the key player in the age-dependent pathological immune-profiles of COVID-19.

The mechanism underlying the significant age-dependent lymphopenia in COVID-19 patients is unknown and is possibly attributed to aging related immunosenescence [2]. Both human and animal studies have shown that CD8^+^ cytotoxic T cells were critical in the mediation of viral clearance in human respiratory syncytial virus and influenza A virus infections [14–18]. In SARS-CoV infections, T cell lymphopenia and a decrease in CD8^+^ T cells are a prominent part of the disease, which may be due to a direct infection of lymphocytes by SARS-CoV or lymphocyte sequestration in the lung [19]. Thus, cytotoxic immunity (particularly for CD8^+^ T cells) may be a key player in the determination of age-dependent antiviral processes in COVID-19 patients. As the older age-groups had more severe and critically ill cases, our data was consistent with previous findings which indicated that CD8^+^ T cells may be an independent predictor for COVID-19 severity [5].

In both SARS-CoV and SARS-CoV-2 infections, proinflammatory cytokines including IL-6 and tumor necrosis factor (TNF)-α were found to be markedly higher in severe cases than in moderate cases [3, 20]. In the present study, the abnormal high IL-6 levels were observed in the older age-groups alone. CRP, which is the first acute-phase protein to be identified during tissue damage or inflammation, age-dependently and linearly increased in the age-groups 39–57y, 58–69y, and 69–79y when compared with the age-group 2–25y. This suggested that the tissue damage or inflammatory marker CRP may be considered as an independent marker associated with the age-dependent disease severity of COVID-19.

There were a few of limitations in this study. First, this retrospective study mainly analyzed the data associated with counts of T cell subsets, B cells and NK cells; the function of these cells and the roles of other immune cells and inflammatory cells remain to be determined. Second, the age-groups 2–25y (n=34), 58–68y (n=41), and 69–79y (n=16) comprised a relatively small number of patients; therefore, caution should be taken with regard to the interpretation of these results, and statistical non-significance may not rule out differences among the different age-groups. Third, age disparities in COVID-19 cases may be explained by other factors associated with differences in the susceptibilities and manifestations of clinical symptoms among the age groups. The present study did not address or identify these differences.

In conclusion, based on an age-COVID-19 incidence-dependent division of chronological age, we found that the SARS-CoV-2 infection induced an age-dependent lymphopenia, particularly an age-dependent decrease in CD8^+^ T cell counts. The CD8^+^ T cell subset is critical in the mediation of viral clearance, and our studies revealed that it may also be a major player in immunosenescence, referred to the age-dependent decline of the immune system. Furthermore, the CRP responses to COVID-19 is also age-dependent. Gaining a deeper comprehension of the age-related factors that can induce age-dependent changes in the immune features and their association with the COVID-19 severities is of importance in the understanding and management of the disease.

## MATERIALS AND METHODS

### Study design and participants

We performed a retrospective review of the medical records of 693 COVID-19 patients admitted to 10 hospitals in Wenzhou City, Zhejiang Province, China as of March 20, 2020. The diagnosis of COVID-19 was made according to the interim guidance of the World Health Organization [21] and confirmed by RNA detection of the 2019-nCoV in the clinical laboratories of hospitals as described previously [11]. Based on the guidelines for diagnosis and management of COVID-19 (7th edition, in Chinese) released by the National Health Commission of China [22], the COVID-19 patients were stratified into mild (with mild symptoms and no sign of pneumonia), moderate (with pneumonia and arterial oxygen partial pressure/fractional inspired oxygen [PaO2/FIO2] >300 mmHg), severe (with pneumonia and respiratory distress, respiratory rate >30 breaths/min; oxygen saturation <93% at rest; and PaO2/FIO2 <300 mmHg) and critically ill (with respiratory failure and a requirement for mechanical ventilation, shock, and complications from other organ failures that required monitoring and treatment in the intensive care unit) groups.

This study was reviewed and approved by the Ethics Committee of Wenzhou Medical University (Ref 2020002). Written informed consent was waived due to the rapid emergence of COVID-19.

Accurate patient age-subgrouping is an important prerequisite for the generation of accurate age-dependent results. If the age boundaries are arbitrarily or roughly defined, the age-related analysis could result in questionable findings. As medical interventions can influence many parameters giving rise to conflicting data, the parameters were assessed only at pre-hospitalization. To further differentiate COVID-19 patients who were previously healthy from those with comorbidities, which showed strong interference in COVID-19 manifestations, data from 447 patients without comorbidities on admission were included in the analysis. Therefore, different age-cohorts were combined with their neighboring age cohorts with similar incidence counts/year together. Accordingly, the five age-groups, 2‒25y, 26‒38y, 39‒57y, 58‒68y, and 69–93y were formed.

### Collection of clinical and laboratory data

We collected epidemiological, demographic, clinical, laboratory, treatment, and outcome data from the electronic medical records. Data were obtained and curated with a customized data collection form. Three physicians (CC, BY, and TL) and a third researcher (SJ) checked all the data and adjudicated any differences in interpretation among the three primary reviewers. Routine blood examinations in patients on admission included complete blood counts (white blood cells, neutrophils, lymphocytes, and platelets), serum biochemical tests (for renal and liver function, creatine kinase, lactate dehydrogenase, myocardial enzymes, CRP, IL-6, IL-2, IL-4, IL-5, IL-10, TNF-α, and IFN-γ). The coagulation tests, prothrombin time, activated partial thromboplastin time, fibrinogen test, and d-dimer test were also performed.

### Flow cytometry analysis

To detect the phenotypic characteristics of the lymphocytes (CD4^+^ and CD8^+^ T-cells, and B-cells and NK cells), samples of ethylenediaminetetraacetic acid anticoagulated peripheral blood (2 mL) were collected from COVID-19 patients before initial treatment and a second sample was collected after 12 days of treatment. Measurements were performed as previously described [6]. Briefly, CD4^+^ and CD8^+^ T-cell, CD19^+^ B-cell, and CD16^+^ CD56^+^ NK-cell staining was performed with the following antibodies: Peridin chlorophyll protein-conjugated anti-human CD3 mAb (BD Biosciences, California, USA), allophycocyanin (APC)-conjugated anti-human CD4 mAb (BD Biosciences), APC/Cy7-conjugated anti-human CD8 mAb (Biolegend, USA), APC-conjugated anti-human CD19 mAb (BD Biosciences), APC-conjugated anti-human CD16, and Brilliant™ Violet 510 (BV-510)-conjugated anti-human CD56 mAb (Biolegend). The gate strategy of CD4^+^ T-cells, CD8^+^ T-cells, B-cells, and NK cells was executed as CD3^+^CD4^+^, CD3^+^CD8^+^, CD3^−^CD19^+^, and CD3^−^CD16^+^/CD56^+^, respectively, and the cells were analyzed using multiple-color flow cytometry on a BD FACS Canto II flow cytometry system (BD Biosciences).

### Statistical analyses

Where appropriate according to the data distribution, the results were reported as means±standard deviations, medians (IQRs), or as categorical variables as numbers and percentages. The distributions were compared using the D'Agostino and Pearson omnibus normality, Shapiro-Wilk normality, and Kolmogorov-Smirnov tests. Categorical variables were shown as frequencies (%). The Mann-Whitney U test, Kruskal-Wallis test, χ^2^ test, Chi-square with a Yates' correction, or Fisher’s exact test was used for nonparametric data where appropriate. Spearman correlation coefficients were performed to determine the associations between variables. A p-value of <0.05 was considered statistically significant. GraphPad Prism 8.0 software (GraphPad Software, Inc., San Diego, CA, USA) was used for the statistical analyses.

### Ethical approval

This study conformed to the ethical guidelines of the 1975 Declaration of Helsinki. The ethics approval has been issued by the Ethics Committee of Wenzhou Medical University (Ref 2020002).
